# Eight coding-complete genomes of human metapneumovirus recovered by virus metagenomics in coastal Kenya, 2021–2024

**DOI:** 10.1128/mra.00065-26

**Published:** 2026-03-16

**Authors:** Samuel K. Mwasya, Dorcas Okanda, Samuel Odoyo, Esther Nyadzua Katama, Clement Lewa, Arnold W. Lambisia, George Githinji, Charles N. Agoti

**Affiliations:** 1 Kenya Medical Research Institute, KEMRI-Wellcome Trust Research Programme 285561 , Kilifi, Kenya; 2 Department of Biochemistry and Biotechnology, Pwani University 270495, Kilifi, Kenya; 3 School of Public Health, Pwani University 270495, Kilifi, Kenya; DOE Joint Genome Institute, Berkeley, California, USA

**Keywords:** human metapneumovirus, hMPV, genomic surveillance, lineages, metagenomics, Kilifi, Kenya

## Abstract

Few human metapneumovirus coding-complete genomes are available from Africa despite significance in optimizing local molecular diagnostics and global phylogeographic analysis. We report eight genomes recovered following nanopore metagenomic sequencing of samples collected in coastal Kenya (2021–2024). These fell into sub-lineages A2b/A2.2.1 (*n* = 3), A2c-wt/A2.2.2 (*n* = 4), and B1 (*n* = 1).

## ANNOUNCEMENT

Human metapneumovirus (hMPV; genus *Metapneumovirus*, family *Pneumoviridae*) is a globally ubiquitous respiratory pathogen that infects persons of all ages, causing severe disease in young children and the elderly (1). A surge of hMPV cases in late 2024 in China raised international concern about an unusually severe epidemic that might spread beyond (2). hMPV genome is a negative-sense, single-stranded RNA molecule (~13.3 kb) encoding nine proteins across eight genes (N, P, M, F, M2, SH, G, and L) (3). Clinical isolates divide into two antigenic lineages (A and B), further subdivided into sub-lineages based on phylogenetic analysis of F and/or G gene. Spatially and temporally representative genomic data are crucial for updating molecular diagnostics, revealing molecular adaptations, and tracking global spread and transmission (4). However, by December 2025, only 3.4% (67/1,969) genomes (>90% completeness) and 4.4% (148/3360) G gene sequence in GenBank originated from Africa. The paucity of hMPV genomic data from Africa impedes full understanding of its local and global epidemiology.

We report eight hMPV genomes from nasopharyngeal/oropharyngeal swabs collected between 2021 and2024, during acute respiratory infection surveillance studies within Kilifi, Kenya (Table 1). Participants were enrolled in outpatient facility-based surveillance, inpatient pediatric pneumonia surveillance at Kilifi County Referral Hospital, and community-based respiratory infection surveillance. Samples identified as hMPV positive (quantitative PCR [qPCR] Ct < 35.0) as previously described (5) underwent metagenomic sequencing. Viral RNA was extracted using QIAamp Viral RNA Mini Kit (QIAGEN), treated with TURBO DNase (37°C, 30 min), and purified with Zymo RNA Clean & Concentrator-5 kit. Sequence-independent single-primer amplification used Sol-A primer (5′-GTTTCCCACTGGAGGATA-N9-3′) for first-strand synthesis with SuperScript IV RT, and second-strand synthesis with Sequenase V2.0 complementary DNA underwent PCR amplification using primers (5′-GTTTCCCACTGGAGGATA-3′) and Q5 polymerase (30 cycles: 94°C 15 s, 50°C 20 s, 68°C 2 min). Amplified products were purified with AMPure XP beads (×0.6 ratio), quantified with Qubit dsDNA HS assay, and libraries prepared using Native Barcoding V14 Kit for GridION sequencing on R10.4.1 flowcells and automated basecalling with Guppy v6.3.9 (https://nanoporetech.com/document/Guppy-protocol).

**TABLE 1:** Sample information, read metrics, and associated links to GenBank and sequence read archive (SRA)

Sample ID	Collection date	Study^*a*^	Clinical status (Ct)^*b*^	Sequence length	GC content (%)	Total reads	hMPV reads	Sub-lineage	GenBank closest hit	Identity (%)	Country, year	GenBank accession	SRA accession
HF001/21	09-Jun-21	HF	S (22.0)	13,292	36.5	1,547,680	101,309	B1	PP947569.1	‍99.6	‍USA, 2020	PX120316	SRR35000059
HF002/23	18-Sep-23	HF	S (24.0)	13,321	36.4	949,219	6,277	A2c-wt/A2.2.2	OL794413.1	‍98.5	Netherlands, 2010	PX120317	SRR35000058
HF003/23	04-Oct-23	HF	S (24.3)	13,325	36.3	834,365	26,876	A2c-wt/A2.2.2	OL794413.1	‍98.5	Netherlands, 2010	PX120318	SRR35000057
HS001/23	30-Nov-23	ResViRe	A (24.6)	13,183	36.5	566,197	3,031	A2c-wt/A2.2.2	OL794413.1	‍98.6	Netherlands, 2010	PX120320	SRR35000056
HF004/24	09-Jan-24	HF	S (23.8)	13,305	36.3	652,679	9,716	A2c-wt/A2.2.2	OL794413.1	‍98.5	Netherlands, 2010	PX120319	SRR35000055
IP001/24	11-Nov-24	IP	S (24.0)	13,324	36.0	2,455,055	1,678	A2b/A2.2.1	PV252964.1	‍99.3	‍USA, 2023	PX120321	SRR35000054
IP002/24	13-Nov-24	IP	S (26.5)	13,321	36.1	1,271,351	7,587	A2b/A2.2.1	PV252964.1	‍99.5	‍USA, 2023	PX120322	SRR35000053
IP003/24	18-Nov-24	IP	S (25.3)	13,322	36.1	758,305	14,296	A2b/A2.2.1	PV252964.1	‍99.4	‍USA, 2023	PX120323	SRR35000052

^*a*^ ResViRe is the community-based study that collected and analyzed respiratory swabs irrespective of symptom status; IP is the inpatient pediatric pneumonia study, with swabs collected from infants admitted to Kilifi County Hospital with respiratory illness; and HF is the outpatient acute respiratory illness study, where swabs were obtained from participants presenting with respiratory illness at health facilities across Kilifi County.

^*b*^ Ct is qPCR cycle threshold; S stands for symptomatic while A stands for asymptomatic.

Bioinformatic analysis removed human reads from raw reads using Kraken2 (v2.0.8-beta) (6) against prebuilt standard-16 database; https://benlangmead.github.io/aws-indexes/k2). *Homo sapiens* reads were excluded and adapters trimmed using Porechop v0.2.4 (7). Contigs were assembled using metaFlye v2.9.6-b1802 (8). Contigs >12 kb and >100× depth underwent BLASTn (9) against custom reference database of representative hMPV sub-lineages (accessions: OL794375.1, OL794442.1, OL794455.1, PP591753.1, PV081663.1, PV353978.1, KJ627399.1, and NC_039199.1) to identify best reference (>90% identity). The best-hit reference for each sample was used for mapping with Minimap2 v2.22-r1101 (10), processed with Samtools v1.13 (11), and consensus sequence generated with iVar v1.3.1 (12). Lineage assignment combined phylogenetics and nextclade (v3.18.1) (https://clades.nextstrain.org/). All tools were run with default parameters unless otherwise specified.

**Fig 1:**
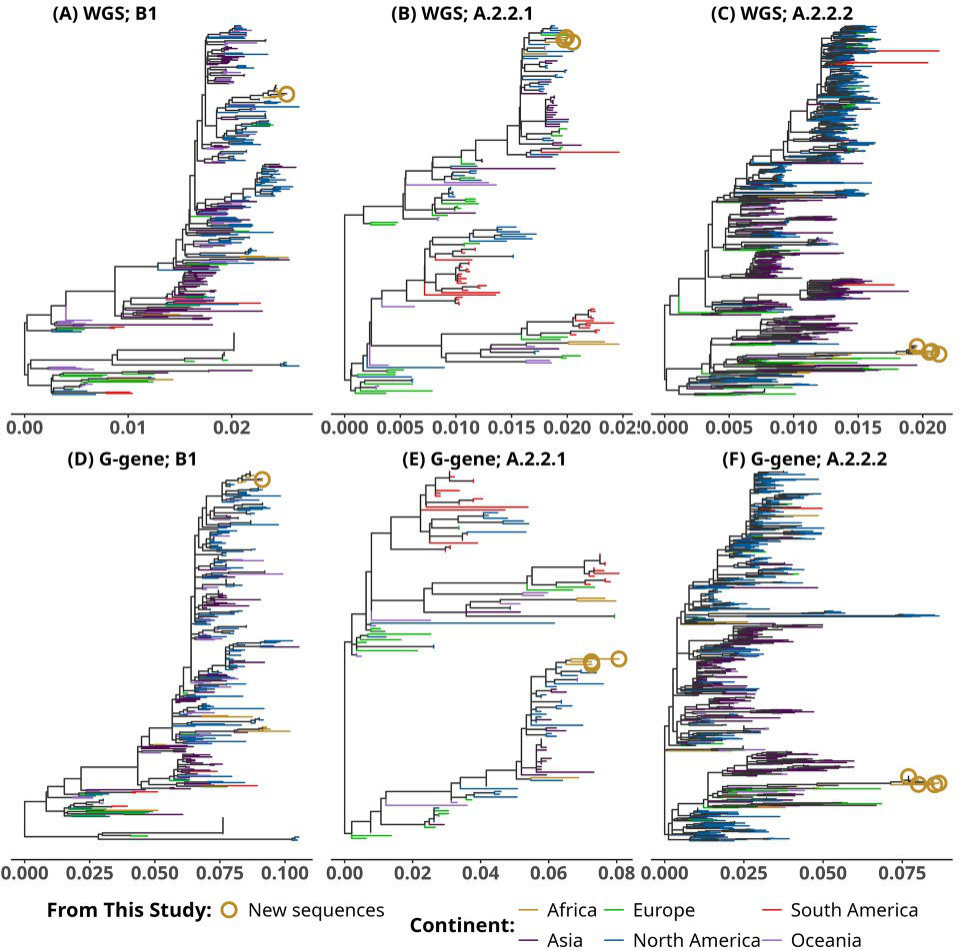
Maximum likelihood (ML) lineage-specific phylogenetic trees of eight hMPV genomes generated in this study, analyzed in the context of global hMPV genomes (>10 kb), and retrieved from GenBank up to 2025. All trees were reconstructed under the HKY+F+R4 nucleotide substitution model on IQ-TREE2 v2.3.5 (13). The top panel shows ML trees inferred from coding-complete genomes, while the bottom panel shows ML trees inferred from G gene sequences. (**A and D**) Lineage B1 (*n* = 324). (**B and E**) Lineage A2b/A2.2.1 (*n* = 158). (**C and F**) Lineage A2c-wt/A2.2.2 (*n* = 911). Tips are colored according to continent of sampling. Sequences generated in this study are indicated by orange circles.

The genomes had a median length of 13,321 bp (interquartile range 13,299–13,323 bp) and >100× coverage (Table 1). Sub-lineage-specific phylogenies, with available GenBank sequences up to 2025, showed our sequences clustering with sequences from other continents, except A2c-wt/A2.2.2 formed a distinct cluster with predominantly African sequences (Fig. 1).

In conclusion, we present eight coding-complete hMPV sequences from coastal Kenya (2021–2024). These data will contribute to characterizing global diversity, molecular diagnostic design, and phylogeographic analyses.

## Data Availability

Consensus hMPV genomes generated in this study were deposited in the GenBank database under the accession numbers PX120316 to PX120323. The raw sequencing reads were deposited in the NCBI Sequence Reads Archive database under the SRA accession number: PRJNA1306485.
